# Inhibition of COX-2 and PI3K/AKT Pathways to Prevent Cancer Metastasis

**DOI:** 10.34172/apb.44038

**Published:** 2025-03-23

**Authors:** Punet Kumar, Sangam Singh

**Affiliations:** ^1^Department of Pharmaceutical Chemistry, Shri Gopichand College of Pharmacy, Dr. A.P.J. Abdul Kalam Technical University, Baghpat, India; ^2^Department of Pharmaceutical Chemistry, Oxford College of Pharmacy, Dr. A.P.J. Abdul Kalam Technical University, Hapur, India

## To the Editor,

 Metastasis of cancer is one of the main reasons for treatment failure and poorer prognosis of patients with metastatic cancers such as non-small cell lung cancer (NSCLC).^1^ Metastasis is central to these pathways, as complex interactions between tumor cells and the host microenvironment are necessary for metastatic spread. Tumor progression, inflammation-driven has been confirmed for a decade, and targeting these pathways in cancer patients has been actively studied as cancer therapy.^2,3^ Cyclooxygenase-2 (COX-2), the principal isoform over-expressed in many cancers, including NSCLC, can exert a pro-inflammatory effect and support tumor growth/migratory capacities by further promoting angiogenesis. COX-2-derived prostaglandins contribute to cancer cell survival, invasiveness, and resistance to apoptosis. Additionally, COX-2 activation triggers downstream signaling pathways, such as PI3K/AKT, which play a crucial role in metastasis and tumor cell survival. COX-2 overexpression in tumor cells also leads to subsequent downstream signaling such as activation of the PI3K/AKT downstream pathway, an essential factor for metastasis and resistance to apoptosis.^4^ Recent studies demonstrate the COX-2 inhibitor, celecoxib, may be one tool for halting tumor progression and metastasis. Inhibitors are effective in the clinic to prevent the metastatic spread and lower the side effects of currently used chemo.^5^ COX-2 inhibitors can target inflammatory aspects but the antimetastatic impact is under-determined, and thus further investigation of therapeutic interventions is necessary as an improvement.^6^ NS398, a COX-2 inhibitor in itself and similarly in combination with either of 2 PI3K/AKT inhibitors LY294002 has been detected to implement a G2/M arrest in WM35 melanoma cells signifying their potential in cancer treatment.^7^ PI3K-AKT inhibitors have also been reported to function as an effectual suppressor of COX-2 inhibitors further lowering cell proliferation and migration in lung cancer cells, synergistically. This may indicate that targeting both pathways represents a more potent therapeutic strategy against metastasis.^8^

 An efficient and unique way might be the design of dual-target inhibitors, that can inhibit two inflammatory pathways in tandem, one targeting COX-2 and the other one the PI3K/AKT pathway. The PI3K/AKT axis is well-known to be essential for the regulation of cell survival, growth, and migration. Its activation when induced by COX-2 signaling significantly contributes to cancer metastasis.^9^ Due to targeting both COX-2 and PI3K/AKT pathways, these dual-target inhibitors, consequently fall on many of other pro-tumorigenic & metastatic processes causing a more holistic strategy to combat cancer metastasis.^10^ Although with considerable potential, no dual-target inhibitors have been developed and translated into clinical practice thus far. The most significant obstacle pertinent to its development is the need to target selectivity and reduce off-target effects.^11^ It requires an in-depth investigation of the pharmacokinetics and safety of these inhibitors to prove efficacy without being patient-unsafe formulations.^12^ Preclinical and clinical work must target to overcome these challenges by further enhancing drug formulations as well as identifying the most appropriate subset of patients for these therapies.^13^ Combining COX-2 inhibitors with PI3K/AKT inhibitors, or developing next-generation dual-target agents, presents a promising strategy for therapeutic advancement. With this approach, it can fortify current cancer therapy, decrease metastasis potential, and provide patients with a superior way to stabilize disease progression.^14^ Further studies are needed to assess the clinical utility of dual-target inhibitors in conjunction with standard-of-care therapy and confirm the validity of how they can be translated to clinical applications.

 Finally, double targeting of COX-2 and PI3K/AKT pathways in inhibition of inflammation-dependent cancer progression may pave new avenues for metastasis reduction on top of clinical benefit in cancer patients. Research focused on dual-target inhibitors could substantially increase the efficacy of cancer therapies and would be an exciting approach to overcoming the challenges underlying metastatic disease.^15^ Continued studies of dual-target inhibitors, possibly supported by approaches to surmount challenges in their translational implementation might lead to major benefits for cancer therapies, and therefore provide a hopeful roadmap for addressing the challenges of metastasis.

 We encourage further investigation in this area to realize the full therapeutic potential of these promising combination strategies.

## Competing Interests

 The authors state that there is no competing/conflict of interest.

## Ethical Approval

 Not applicable.
